# State of the Globe: Evaluating the Existence of Extended Spectrum Beta Lactamases

**DOI:** 10.4103/0974-777X.56245

**Published:** 2009

**Authors:** Stephen E Mshana

**Affiliations:** *Department of Microbiology, Weill Bugando Medical, College of Health Sciences, BOX 1464 Mwanza, Tanzania*

Beta-lactam antibiotics remain the most widely used antibiotic worldwide. A major threat for these compounds is b-lactamases; several b-lactamases have been identified (SHV, TEM, CTX-M etc). Most of these are extended, able to hydrolyze broad-spectrum cephalosporins and aztreonam.[1]

The study on ESBL in this issue was designed to estimate the magnitude of extended spectrum beta-lactamase producers and evaluate ESBL screening method against confirmatory method.[2] It was conducted at a tertiary health hospital in Sikkim, India, where data on ESBL is limited. About 1489 specimens, over a period of 12 months, were collected from patients suspected with nosocomial infections. Specimens were processed according to the standard operative procedures. A total of 258 isolates, from 258 specimens, with significant growth were included in the analysis. The overall prevalence of ESBL among Gram negative isolates was 76/258 (29.4%). *Escherichia coli* was the most recovered isolate and 34/130 (26.2%) were ESBL producers. About 15/35 (42.8%) of *Klebsiella pneumoniae* and 15/46 (32%) of *Pseudomonas aeruginosa* were ESBL producers. Other Gram negative (*Proteus mirabili*s, *Enterobacter cloacae*, *M. Morganii* and *C. freundii*) had ESBL rate of 12/27 (44.4%).

Considering the epidemiology of ESBL isolates and nosocomial infection, the results were similar to many other studies in developing and developed countries.[3] The prevalence of ESBL in the study area is high and poses a threat to treatment of serious infections due to these isolates. Most ESBL-producing isolates in the study were significantly resistant to gentamicin, SXT, tetracycline and ciprofloxacin. Under such circumstances, the only treatment choices are carbepenems or tigecycline which are expensive and often not available in most centers in the developing countries.

ESBL genes are carried in large plasmids, sometimes associated with multiple resistance genes. This could explain why most ESBL producing organisms in the study were significantly resistant to multiple antibiotics.[4] With the exception of *Pseudomonas spp,* all ESBL isolates from the study were sensitive to carbepenems. These drugs have been considered drugs of choice for ESBL isolates. There are increasing reports of *Klebsiella pneumoniae* ESBL isolate resistance to carbepenems. This makes the treatment option of these isolates difficult in developing countries.[5] Most *Klebsiella pneumonia* are ESBL producers; almost all non- ESBL producing *Klebsiella pneumoniae* isolates have chromosomally mediated SHV-1 lactamase.[6] This could also explain why 100% of *Klebsiella pneumoniae* are resistant to ampicillin with a significant amount being ESBL producers. A significant proportion of other enteric bacteria like *Proteus spp*, *Morganella morganii, Enterobacter spp*, *Citrobacter spp* and *Pseudomonas spp* were found to produce ESBL at similar rates with results obtained in other studies elsewhere.[3]

The study also evaluated double disc-diffusion test as a screening method to identify ESBL isolates. This was crucial because cheap and reliable methods are needed in microbiology laboratories in many developing countries. The use of double disc synergy (Ceftazidime 30μg and cefotaxime 30μg discs) placed equidistant from the amoxycillin/clavunate 20/10 μg) disc; has been found to be cheaper and reliable with results comparable to confirmatory test.[3] As similar results were observed in the study it is recommended for routine use in the clinical microbiology laboratory. It is important for any modification in laboratory testing to keep in mind developing countries, to be acceptable worldwide. Use of double disc synergy test as a routine in clinical laboratory is an important intervention to detect and report ESBL producers to implement contact precautions and prescribe appropriate antibiotics.

ESBL isolates are prevalent in developing countries and multiple resistant to gentamicin, ciprofloxacin, tetracycline and sulphamethaxazole/trimethoprim. This poses a challenge to treatment of patients infected with ESBL isolates. Routine detection of ESBL isolates and proper control measures are urgently needed for appropriate management.

## References

[1] Livermore DM (2008). Defining an extended β spectrum-lactamase. Clin Microbiol Infect.

[2] Tsering DC, Das S, Adhiakari L, Pal R, Singh TS (2009). Extended spectrum beta-lactamase detection in gram-negative bacilli of nosocomial origin. J Global Infect Dis.

[3] Paterson DL, Bonomo RA (2005). Extended-spectrum β-lactamases: A clinical update. Clin Microbiol Rev.

[4] Boyd DA, Tyler S, Christianson S, McGee A, Muller MP, Willey BM (2004). Complete nucleotide sequence of a 92-kilobase plasmid harboring the CTX-M-15 extended-spectrum beta-lactamase involved in an outbreak in long-term-care facilities in Toronto, Canada. Antimicrob Agent Chemother.

[5] Grobner S, Linke D, Schutz W, Fladerer C, Madlung J, Autenrieth IB (2009). Emergence of carbapenem-non-susceptible extended-spectrum b-lactamase-producing Klebsiella pneumoniae isolates at the university hospital of Tu¨ bingen, Germany. J Med Microbiol.

[6] Babin GS, Livermore DM (2000). Are SHV beta-lactamases universal in Klebsiella pneumoniae?. Antimicrob Agents Chemother.

